# Crystal structure of 4-oxo-4*H*-chromene-3-carb­oxy­lic acid

**DOI:** 10.1107/S2056989015013456

**Published:** 2015-07-17

**Authors:** Yoshinobu Ishikawa

**Affiliations:** a School of Pharmaceutical Sciences, University of Shizuoka, 52-1 Yada, Suruga-ku, Shizuoka 422-8526, Japan

**Keywords:** crystal structure, chromone, hydrogen bonding, stacking inter­action

## Abstract

In the title compound, C_10_H_6_O_4_, also known as 3-carb­oxy­chromone, the non-H atoms of the chromone ring are essentially coplanar (r.m.s. deviation = 0.0057 Å), with the maximum deviation from their least-squares plane [0.011 (2) Å] being for a pyran C atom. The dihedral angle between the fused ring and plane of the carb­oxy group is 3.06 (2)°. An intra­molecular hydrogen bond is formed between the ring carbonyl O atom and the carb­oxy O—H atom, closing an *S*(6) loop. In the crystal, mol­ecules are assembled by stacking inter­actions [centroid–centroid distance between the benzene and pyran rings = 3.844 (3) Å] and C—H⋯O hydrogen bonds, generating a three-dimensional network. Short contacts are also observed between the carb­oxy O and C atoms [C=O⋯C=O = 3.002 (3) Å].

## Related literature   

For the biological activities of the title compound and its related compounds, see: Alcaro *et al.* (2010 ▸); Gaspar *et al.* (2012 ▸); Legoabe *et al.* (2012 ▸); Papaneophytou *et al.* (2015 ▸). For the synthesis of the title compound, see: Helguera *et al.* (2013 ▸).
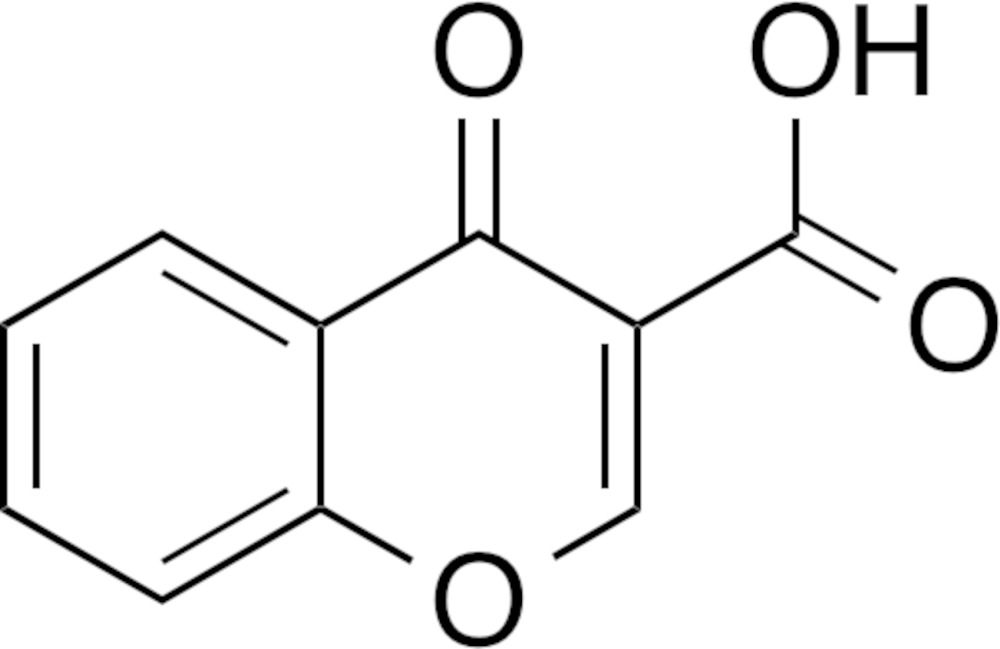


## Experimental   

### Crystal data  

C_10_H_6_O_4_
*M* *_r_* = 190.15
Monoclinic, 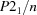

*a* = 18.017 (8) Å
*b* = 5.549 (3) Å
*c* = 8.017 (5) Å
β = 92.49 (4)°
*V* = 800.8 (8) Å^3^
*Z* = 4
Mo *K*α radiation
μ = 0.12 mm^−1^
*T* = 100 K
0.37 × 0.35 × 0.05 mm

### Data collection  

Rigaku AFC-7R diffractometer
2228 measured reflections
1831 independent reflections
1183 reflections with *F* ^2^ > 2.0σ(*F* ^2^)
*R*_int_ = 0.098
3 standard reflections every 150 reflections intensity decay: −1.6%

### Refinement  

*R*[*F* ^2^ > 2σ(*F* ^2^)] = 0.047
*wR*(*F* ^2^) = 0.124
*S* = 1.04
1831 reflections
128 parameters
H-atom parameters constrained
Δρ_max_ = 0.24 e Å^−3^
Δρ_min_ = −0.24 e Å^−3^

### 

Data collection: *WinAFC Diffractometer Control Software* (Rigaku, 1999 ▸); cell refinement: *WinAFC Diffractometer Control Software*; data reduction: *WinAFC Diffractometer Control Software*; program(s) used to solve structure: *CrystalStructure* (Rigaku, 2010 ▸); program(s) used to refine structure: *SHELXL97* (Sheldrick, 2008 ▸); molecular graphics: *CrystalStructure* (Rigaku, 2010 ▸); software used to prepare material for publication: *CrystalStructure*.

## Supplementary Material

Crystal structure: contains datablock(s) General, I. DOI: 10.1107/S2056989015013456/tk5375sup1.cif

Structure factors: contains datablock(s) I. DOI: 10.1107/S2056989015013456/tk5375Isup2.hkl

Supporting information file. DOI: 10.1107/S2056989015013456/tk5375Isup3.cml

**Fig. 1.**
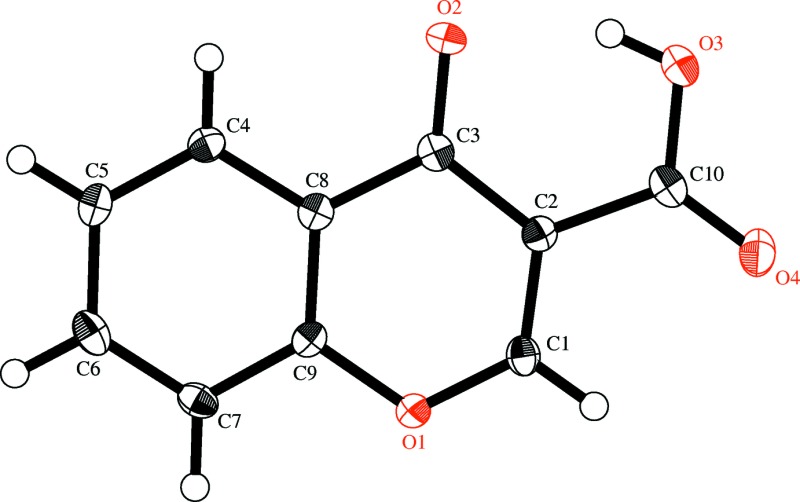
The molecular structure of the title compound, with displacement ellipsoids drawn at the 50% probability level. Hydrogen atoms are shown as small spheres of arbitrary radius.

. DOI: 10.1107/S2056989015013456/tk5375fig1.tif
The mol­ecular structure of the title compound, with displacement ellipsoids drawn at the 50% probability level. Hydrogen atoms are shown as small spheres of arbitrary radius.

**Fig. 2.**
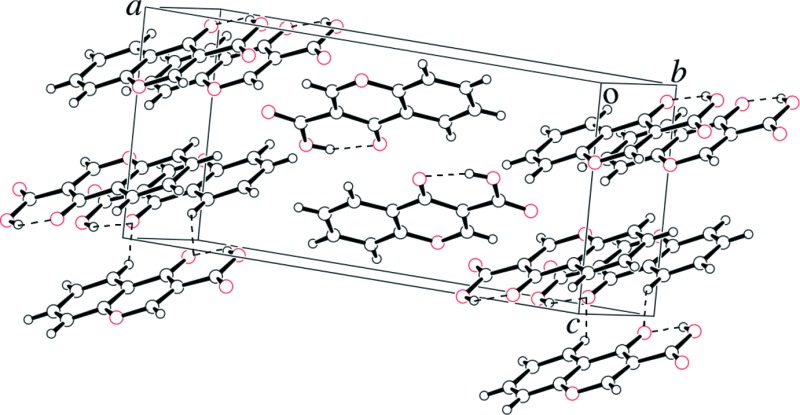
A view of the intermolecular interactions of the title compound. Intramolecular O—H···O and intermolecular C—H···O hydrogen bonds are represented as dashed lines.

. DOI: 10.1107/S2056989015013456/tk5375fig2.tif
A view of the inter­molecular inter­actions of the title compound. Intra­molecular O—H⋯O and inter­molecular C—H⋯O hydrogen bonds are represented as dashed lines.

CCDC reference: 1412580

Additional supporting information:  crystallographic information; 3D view; checkCIF report

## Figures and Tables

**Table 1:** Hydrogen-bond geometry (, )

*D*H*A*	*D*H	H*A*	*D* *A*	*D*H*A*
O3H6O2	0.84	1.79	2.570(3)	153
C4H2O2^i^	0.95	2.45	3.298(3)	149
C1H1O4^ii^	0.95	2.41	3.346(3)	168
C6H4O4^iii^	0.95	2.45	3.232(3)	140

Symmetry codes: (i) 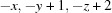; (ii) 
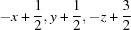
; (iii) 
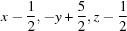
.

## supplementary crystallographic information

### S1. Comment

The title compound (3-carboxychromone) and its derivatives are reported as monoamine oxidase inhibitors (Alcaro *et al.*, 2010; Legoabe *et al.*, 2012), A_3_ adenosine receptor ligands (Gaspar *et al.*, 2012) and tumor necrosis factor inhibitors (Papaneophytou *et al.*, 2015).

The mean deviation of the least-square planes for the non-H atoms of the chromone ring is 0.0057 Å, and the largest deviation is 0.011 (2) Å for C2. These mean that these atoms are essentially coplanar (Fig. 1). The dihedral angle between the fused-ring and carboxy plane is 3.06 (2)°. Intramolecular hydrogen bond is formed between the α,β-unsaturated carbonyl O atom and the carboxy O—H atom.

In the crystal packing, the molecules are assembled by stacking interactions [centroid–centroid distance between the benzene and pyran rings of the 4*H*-chromene units = 3.844 (3) Å] and C–H···O hydrogen bonds, as shown in Fig. 2. Shorter contacts than the sum of van der Waals radii are observed between the carboxy O4 and C10^i^ atoms [O4···C10^i^ = 3.002 (3) Å, i: –*x* + 1, *y* + 1/2, –*z* + 3/2].

### S2. Experimental

The title compound was synthesized from 3-formylchromone according to the literature method (Helguera *et al.* 2013). Single crystals suitable for X-ray diffraction were obtained by slow evaporation of an acetone solution of the title compound at room temperature.

### S3. Refinement

All hydrogen atoms were placed in geometric positions [C–H 0.95 Å and O–H 0.84 Å], and refined using a riding model with *U*_iso_(H) = 1.2*U*_eq_ of the parent atoms.

### Crystal data

**Table:**

C_10_H_6_O_4_	*F*(000) = 392.00
*M**_r_* = 190.15	*D*_x_ = 1.577 Mg m^−^^3^
Monoclinic, *P*2_1_/*n*	Mo *K*α radiation, λ = 0.71069 Å
Hall symbol: -P 2yn	Cell parameters from 25 reflections
*a* = 18.017 (8) Å	θ = 15.0–17.3°
*b* = 5.549 (3) Å	µ = 0.12 mm^−^^1^
*c* = 8.017 (5) Å	*T* = 100 K
β = 92.49 (4)°	Plate, colourless
*V* = 800.8 (8) Å^3^	0.37 × 0.35 × 0.05 mm
*Z* = 4	

### Data collection

**Table:**

Rigaku AFC-7R diffractometer	θ_max_ = 27.5°
ω–2θ scans	*h* = −23→23
2228 measured reflections	*k* = 0→7
1831 independent reflections	*l* = −10→5
1183 reflections with *F*^2^ > 2.0σ(*F*^2^)	3 standard reflections every 150 reflections
*R*_int_ = 0.098	intensity decay: −1.6%

### Refinement

**Table:**

Refinement on *F*^2^	Secondary atom site location: difference Fourier map
*R*[*F*^2^ > 2σ(*F*^2^)] = 0.047	Hydrogen site location: inferred from neighbouring sites
*wR*(*F*^2^) = 0.124	H-atom parameters constrained
*S* = 1.04	*w* = 1/[σ^2^(*F*_o_^2^) + (0.0605*P*)^2^ + 0.0457*P*] where *P* = (*F*_o_^2^ + 2*F*_c_^2^)/3
1831 reflections	(Δ/σ)_max_ < 0.001
128 parameters	Δρ_max_ = 0.24 e Å^−^^3^
0 restraints	Δρ_min_ = −0.24 e Å^−^^3^
Primary atom site location: structure-invariant direct methods	

### Special details

**Table:**

Refinement. Refinement was performed using all reflections. The weighted *R*-factor (*wR*) and goodness of fit (*S*) are based on *F*^2^. *R*-factor (gt) are based on *F*. The threshold expression of *F*^2^ > 2.0 *σ*(*F*^2^) is used only for calculating *R*-factor (gt).

### Fractional atomic coordinates and isotropic or equivalent isotropic displacement parameters (Å^2^)

**Table:**

	*x*	*y*	*z*	*U*_iso_*/*U*_eq_	
O1	0.05120 (7)	1.2950 (3)	0.66885 (17)	0.0166 (4)	
O2	0.09613 (7)	0.6952 (3)	0.95238 (17)	0.0190 (4)	
O3	0.22945 (8)	0.8524 (3)	1.00156 (19)	0.0230 (4)	
O4	0.26339 (8)	1.1851 (3)	0.87416 (19)	0.0233 (4)	
C1	0.11926 (10)	1.2529 (4)	0.7381 (3)	0.0165 (5)	
C2	0.13725 (10)	1.0587 (4)	0.8324 (3)	0.0147 (4)	
C3	0.08209 (10)	0.8780 (4)	0.8660 (3)	0.0145 (4)	
C4	−0.05085 (10)	0.7609 (4)	0.8086 (3)	0.0158 (4)	
C5	−0.11971 (11)	0.8094 (4)	0.7329 (3)	0.0184 (5)	
C6	−0.13077 (11)	1.0194 (4)	0.6379 (3)	0.0192 (5)	
C7	−0.07373 (11)	1.1805 (4)	0.6183 (3)	0.0167 (4)	
C8	0.00821 (10)	0.9221 (4)	0.7888 (3)	0.0150 (4)	
C9	−0.00452 (10)	1.1301 (4)	0.6937 (3)	0.0143 (4)	
C10	0.21539 (11)	1.0393 (4)	0.9027 (3)	0.0171 (5)	
H1	0.1572	1.3676	0.7193	0.0197*	
H2	−0.0435	0.6191	0.8737	0.0190*	
H3	−0.1596	0.6999	0.7454	0.0221*	
H4	−0.1783	1.0509	0.5864	0.0231*	
H5	−0.0815	1.3234	0.5546	0.0200*	
H6	0.1909	0.7684	1.0077	0.0276*	

### Atomic displacement parameters (Å^2^)

**Table:**

	*U*^11^	*U*^22^	*U*^33^	*U*^12^	*U*^13^	*U*^23^
O1	0.0147 (7)	0.0167 (7)	0.0185 (7)	−0.0005 (6)	0.0009 (6)	0.0031 (6)
O2	0.0196 (7)	0.0168 (7)	0.0205 (8)	0.0020 (6)	−0.0015 (6)	0.0056 (6)
O3	0.0172 (7)	0.0263 (9)	0.0251 (8)	0.0013 (7)	−0.0030 (6)	0.0042 (7)
O4	0.0167 (7)	0.0240 (8)	0.0290 (9)	−0.0021 (7)	−0.0005 (6)	−0.0028 (7)
C1	0.0129 (9)	0.0190 (10)	0.0176 (10)	0.0012 (8)	0.0020 (8)	−0.0018 (8)
C2	0.0147 (9)	0.0156 (10)	0.0140 (9)	0.0006 (8)	0.0026 (8)	−0.0008 (8)
C3	0.0164 (9)	0.0154 (10)	0.0118 (9)	0.0013 (8)	0.0015 (7)	−0.0044 (8)
C4	0.0174 (9)	0.0144 (10)	0.0156 (10)	−0.0001 (8)	0.0017 (8)	−0.0006 (8)
C5	0.0153 (9)	0.0201 (10)	0.0200 (10)	−0.0037 (8)	0.0018 (8)	−0.0022 (9)
C6	0.0165 (10)	0.0250 (11)	0.0160 (10)	0.0046 (9)	−0.0018 (8)	−0.0042 (9)
C7	0.0197 (9)	0.0155 (10)	0.0147 (9)	0.0043 (8)	−0.0009 (8)	−0.0011 (8)
C8	0.0159 (9)	0.0154 (10)	0.0139 (9)	0.0002 (8)	0.0020 (7)	−0.0022 (8)
C9	0.0154 (9)	0.0143 (10)	0.0135 (9)	−0.0013 (8)	0.0026 (7)	−0.0015 (8)
C10	0.0167 (10)	0.0203 (11)	0.0141 (9)	0.0036 (8)	−0.0004 (8)	−0.0041 (9)

### Geometric parameters (Å, º)

**Table:**

O1—C1	1.344 (3)	C5—C6	1.402 (3)
O1—C9	1.379 (3)	C6—C7	1.377 (3)
O2—C3	1.248 (3)	C7—C9	1.390 (3)
O3—C10	1.323 (3)	C8—C9	1.397 (3)
O4—C10	1.213 (3)	O3—H6	0.840
C1—C2	1.348 (3)	C1—H1	0.950
C2—C3	1.445 (3)	C4—H2	0.950
C2—C10	1.497 (3)	C5—H3	0.950
C3—C8	1.464 (3)	C6—H4	0.950
C4—C5	1.384 (3)	C7—H5	0.950
C4—C8	1.404 (3)		
			
O1···C3	2.843 (3)	H3···H4	2.3436
O2···O3	2.570 (3)	H4···H5	2.3300
O2···C1	3.573 (3)	O1···H2^i^	3.0150
O2···C4	2.865 (3)	O1···H5^xii^	2.8421
O2···C10	2.915 (3)	O2···H1^iii^	2.8607
O3···C3	2.828 (3)	O2···H2^iv^	2.4493
O4···C1	2.798 (3)	O2···H3^iv^	3.4273
C1···C7	3.589 (3)	O3···H1^vi^	2.7625
C1···C8	2.758 (3)	O3···H3^v^	3.4773
C2···C9	2.770 (3)	O3···H3^vii^	2.7495
C4···C7	2.804 (3)	O3···H4^v^	3.5090
C5···C9	2.762 (3)	O3···H4^vii^	2.8526
C6···C8	2.786 (3)	O4···H1^vi^	2.4100
O1···O2^i^	3.255 (3)	O4···H4^ix^	2.4481
O1···C4^i^	3.391 (3)	O4···H5^ix^	3.0909
O1···C5^ii^	3.547 (3)	O4···H6^viii^	3.2381
O1···C6^ii^	3.384 (3)	C1···H2^v^	3.5240
O1···C7^ii^	3.537 (3)	C1···H4^ii^	3.3137
O2···O1^iii^	3.255 (3)	C1···H5^xii^	3.3703
O2···C1^iii^	3.035 (3)	C2···H2^v^	3.4555
O2···C4^iv^	3.298 (3)	C2···H4^ii^	3.5224
O2···C7^v^	3.550 (3)	C3···H1^iii^	3.3721
O2···C8^v^	3.562 (3)	C3···H2^iv^	3.5458
O2···C9^v^	3.482 (3)	C3···H2^v^	3.5707
O3···O4^vi^	3.160 (3)	C3···H5^ii^	3.5517
O3···C1^vi^	3.447 (3)	C4···H5^iii^	3.2013
O3···C5^v^	3.511 (3)	C5···H5^iii^	3.1424
O3···C5^vii^	3.346 (3)	C5···H6^v^	3.4189
O3···C6^v^	3.531 (3)	C6···H1^ii^	3.5945
O3···C6^vii^	3.399 (3)	C6···H6^v^	3.3010
O4···O3^viii^	3.160 (3)	C7···H2^i^	3.2116
O4···O4^vi^	3.437 (3)	C7···H3^i^	3.4460
O4···O4^viii^	3.437 (3)	C8···H5^ii^	3.3906
O4···C1^vi^	3.346 (3)	C9···H2^i^	3.1673
O4···C2^viii^	3.241 (3)	C10···H1^vi^	2.7081
O4···C6^ix^	3.232 (3)	C10···H3^v^	3.3630
O4···C7^ix^	3.536 (3)	C10···H4^ix^	3.2814
O4···C10^viii^	3.002 (3)	H1···O2^i^	2.8607
C1···O2^i^	3.035 (3)	H1···O3^viii^	2.7625
C1···O3^viii^	3.447 (3)	H1···O4^viii^	2.4100
C1···O4^viii^	3.346 (3)	H1···C3^i^	3.3721
C1···C6^ii^	3.386 (4)	H1···C6^ii^	3.5945
C2···O4^vi^	3.241 (3)	H1···C10^viii^	2.7081
C2···C4^v^	3.478 (4)	H1···H4^ii^	3.4096
C2···C5^v^	3.588 (4)	H1···H5^xii^	3.0597
C3···C4^v^	3.356 (3)	H1···H6^i^	3.2466
C3···C8^v^	3.454 (3)	H1···H6^viii^	3.3979
C4···O1^iii^	3.391 (3)	H2···O1^iii^	3.0150
C4···O2^iv^	3.298 (3)	H2···O2^iv^	2.4493
C4···C2^v^	3.478 (4)	H2···C1^v^	3.5240
C4···C3^v^	3.356 (3)	H2···C2^v^	3.4555
C4···C7^iii^	3.580 (4)	H2···C3^iv^	3.5458
C5···O1^ii^	3.547 (3)	H2···C3^v^	3.5707
C5···O3^v^	3.511 (3)	H2···C7^iii^	3.2116
C5···O3^x^	3.346 (3)	H2···C9^iii^	3.1673
C5···C2^v^	3.588 (4)	H2···H2^iv^	2.8347
C5···C10^v^	3.556 (4)	H2···H5^iii^	3.0909
C6···O1^ii^	3.384 (3)	H2···H6^iv^	3.5767
C6···O3^v^	3.531 (3)	H3···O2^iv^	3.4273
C6···O3^x^	3.399 (3)	H3···O3^v^	3.4773
C6···O4^xi^	3.232 (3)	H3···O3^x^	2.7495
C6···C1^ii^	3.386 (4)	H3···C7^iii^	3.4460
C7···O1^ii^	3.537 (3)	H3···C10^v^	3.3630
C7···O2^v^	3.550 (3)	H3···H4^xiii^	3.3726
C7···O4^xi^	3.536 (3)	H3···H5^iii^	2.9785
C7···C4^i^	3.580 (4)	H3···H6^iv^	3.3289
C7···C8^ii^	3.565 (4)	H3···H6^x^	3.2355
C7···C9^ii^	3.394 (4)	H4···O3^v^	3.5090
C8···O2^v^	3.562 (3)	H4···O3^x^	2.8526
C8···C3^v^	3.454 (3)	H4···O4^xi^	2.4481
C8···C7^ii^	3.565 (4)	H4···C1^ii^	3.3137
C8···C8^v^	3.519 (4)	H4···C2^ii^	3.5224
C9···O2^v^	3.482 (3)	H4···C10^xi^	3.2814
C9···C7^ii^	3.394 (4)	H4···H1^ii^	3.4096
C9···C9^ii^	3.434 (4)	H4···H3^xiv^	3.3726
C10···O4^vi^	3.002 (3)	H4···H6^v^	3.4215
C10···C5^v^	3.556 (4)	H4···H6^x^	2.9941
O1···H5	2.5280	H5···O1^xii^	2.8421
O2···H2	2.6023	H5···O4^xi^	3.0909
O2···H6	1.7919	H5···C1^xii^	3.3703
O4···H1	2.4544	H5···C3^ii^	3.5517
O4···H6	2.8847	H5···C4^i^	3.2013
C2···H6	2.3211	H5···C5^i^	3.1424
C3···H1	3.2756	H5···C8^ii^	3.3906
C3···H2	2.6839	H5···H1^xii^	3.0597
C3···H6	2.3046	H5···H2^i^	3.0909
C4···H4	3.2675	H5···H3^i^	2.9785
C5···H5	3.2769	H6···O4^vi^	3.2381
C6···H2	3.2735	H6···C5^v^	3.4189
C7···H3	3.2685	H6···C6^v^	3.3010
C8···H3	3.2704	H6···H1^iii^	3.2466
C8···H5	3.2912	H6···H1^vi^	3.3979
C9···H1	3.1956	H6···H2^iv^	3.5767
C9···H2	3.2716	H6···H3^iv^	3.3289
C9···H4	3.2400	H6···H3^vii^	3.2355
C10···H1	2.5390	H6···H4^v^	3.4215
H2···H3	2.3338	H6···H4^vii^	2.9941
			
C1—O1—C9	118.80 (16)	O1—C9—C8	121.58 (17)
O1—C1—C2	124.29 (18)	C7—C9—C8	121.78 (18)
C1—C2—C3	120.57 (17)	O3—C10—O4	121.07 (19)
C1—C2—C10	117.90 (18)	O3—C10—C2	115.74 (18)
C3—C2—C10	121.53 (17)	O4—C10—C2	123.19 (19)
O2—C3—C2	122.87 (17)	C10—O3—H6	109.466
O2—C3—C8	121.96 (17)	O1—C1—H1	117.850
C2—C3—C8	115.17 (17)	C2—C1—H1	117.864
C5—C4—C8	119.74 (19)	C5—C4—H2	120.128
C4—C5—C6	120.23 (19)	C8—C4—H2	120.133
C5—C6—C7	120.86 (19)	C4—C5—H3	119.885
C6—C7—C9	118.65 (19)	C6—C5—H3	119.886
C3—C8—C4	121.68 (18)	C5—C6—H4	119.563
C3—C8—C9	119.59 (17)	C7—C6—H4	119.572
C4—C8—C9	118.73 (17)	C6—C7—H5	120.665
O1—C9—C7	116.64 (17)	C9—C7—H5	120.682
			
C1—O1—C9—C7	−179.46 (15)	C5—C4—C8—C9	0.6 (3)
C1—O1—C9—C8	0.1 (3)	C8—C4—C5—C6	−0.5 (3)
C9—O1—C1—C2	−0.4 (3)	C8—C4—C5—H3	179.5
C9—O1—C1—H1	179.6	H2—C4—C5—C6	179.5
H6—O3—C10—O4	−179.4	H2—C4—C5—H3	−0.5
H6—O3—C10—C2	1.5	H2—C4—C8—C3	0.2
O1—C1—C2—C3	−0.1 (3)	H2—C4—C8—C9	−179.4
O1—C1—C2—C10	−179.20 (16)	C4—C5—C6—C7	−0.1 (3)
H1—C1—C2—C3	179.9	C4—C5—C6—H4	180.0
H1—C1—C2—C10	0.8	H3—C5—C6—C7	180.0
C1—C2—C3—O2	179.94 (17)	H3—C5—C6—H4	−0.0
C1—C2—C3—C8	0.9 (3)	C5—C6—C7—C9	0.5 (3)
C1—C2—C10—O3	176.58 (17)	C5—C6—C7—H5	−179.5
C1—C2—C10—O4	−2.5 (3)	H4—C6—C7—C9	−179.5
C3—C2—C10—O3	−2.5 (3)	H4—C6—C7—H5	0.5
C3—C2—C10—O4	178.41 (17)	C6—C7—C9—O1	179.18 (17)
C10—C2—C3—O2	−1.0 (3)	C6—C7—C9—C8	−0.4 (3)
C10—C2—C3—C8	179.91 (15)	H5—C7—C9—O1	−0.8
O2—C3—C8—C4	0.2 (3)	H5—C7—C9—C8	179.6
O2—C3—C8—C9	179.82 (16)	C3—C8—C9—O1	0.6 (3)
C2—C3—C8—C4	179.26 (15)	C3—C8—C9—C7	−179.78 (16)
C2—C3—C8—C9	−1.1 (3)	C4—C8—C9—O1	−179.71 (16)
C5—C4—C8—C3	−179.76 (16)	C4—C8—C9—C7	−0.1 (3)

Symmetry codes: (i) *x*, *y*+1, *z*; (ii) −*x*, −*y*+2, −*z*+1; (iii) *x*, *y*−1, *z*; (iv) −*x*, −*y*+1, −*z*+2; (v) −*x*, −*y*+2, −*z*+2; (vi) −*x*+1/2, *y*−1/2, −*z*+3/2; (vii) *x*+1/2, −*y*+3/2, *z*+1/2; (viii) −*x*+1/2, *y*+1/2, −*z*+3/2; (ix) *x*+1/2, −*y*+5/2, *z*+1/2; (x) *x*−1/2, −*y*+3/2, *z*−1/2; (xi) *x*−1/2, −*y*+5/2, *z*−1/2; (xii) −*x*, −*y*+3, −*z*+1; (xiii) −*x*−1/2, *y*−1/2, −*z*+3/2; (xiv) −*x*−1/2, *y*+1/2, −*z*+3/2.

### Hydrogen-bond geometry (Å, º)

**Table:**

*D*—H···*A*	*D*—H	H···*A*	*D*···*A*	*D*—H···*A*
O3—H6···O2	0.84	1.79	2.570 (3)	153
C4—H2···O2^iv^	0.95	2.45	3.298 (3)	149
C1—H1···O4^viii^	0.95	2.41	3.346 (3)	168
C6—H4···O4^xi^	0.95	2.45	3.232 (3)	140

Symmetry codes: (iv) −*x*, −*y*+1, −*z*+2; (viii) −*x*+1/2, *y*+1/2, −*z*+3/2; (xi) *x*−1/2, −*y*+5/2, *z*−1/2.
