# Supplementary figures and images for: Crystal structure of 4-oxo-4H-chromene-3-carb­oxy­lic acid
Source: Acta Crystallogr E Crystallogr Commun. 2015 Jul 17;71(Pt 8):o580–1. doi: 10.1107/S2056989015013456 (PMC4571407; doi:10.1107/S2056989015013456)

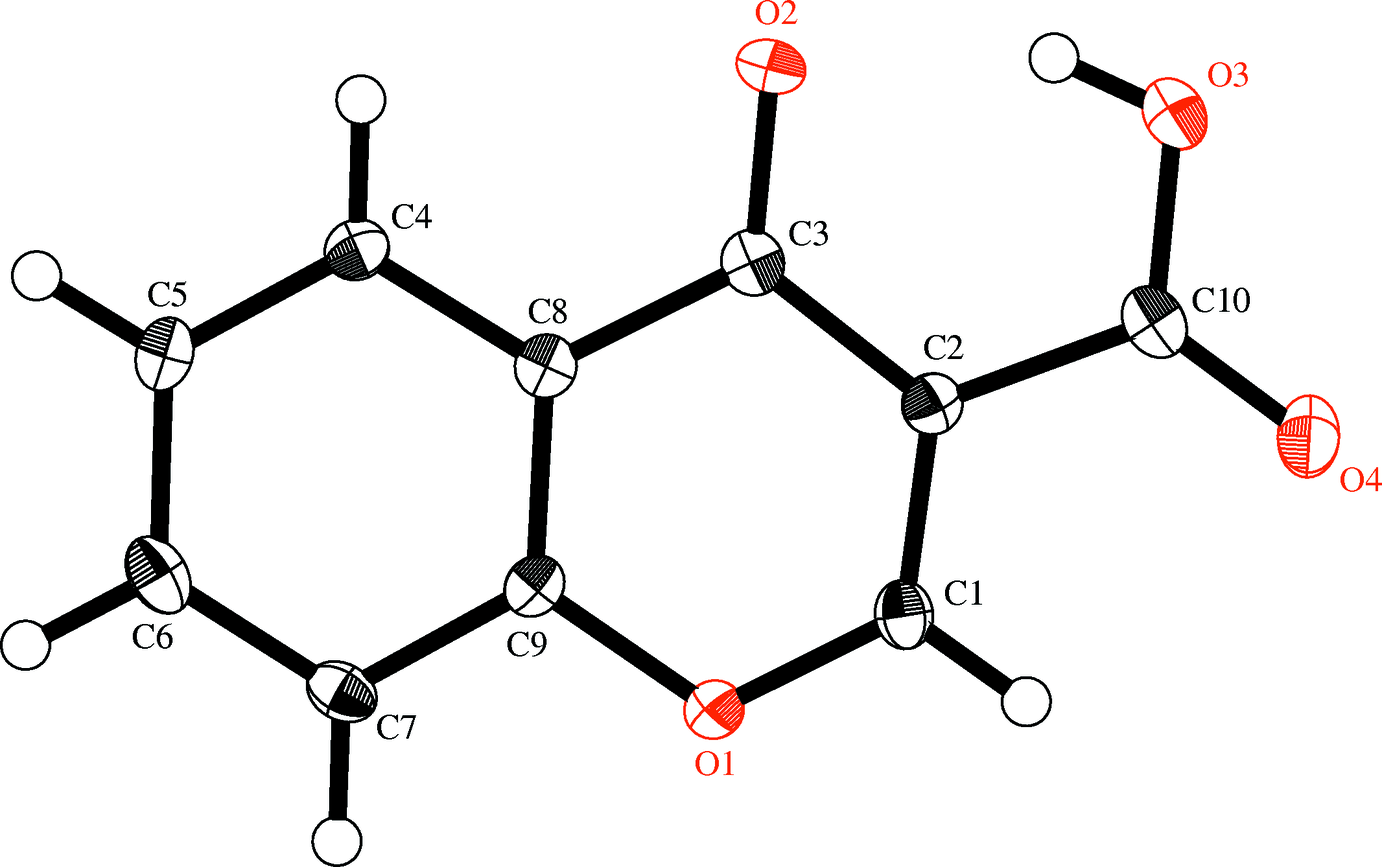

Supplement: Supplementary file 4 [file e-71-0o580-fig1.tif]

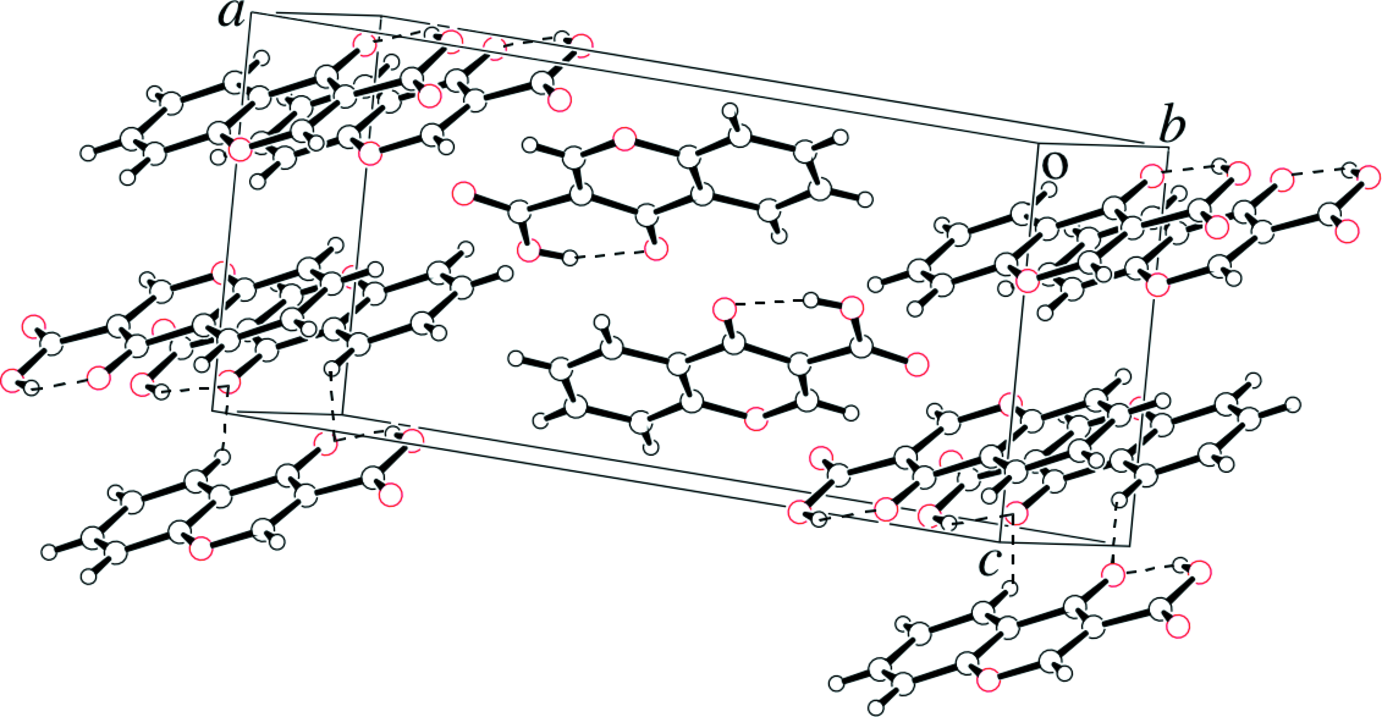

Supplement: Supplementary file 5 [file e-71-0o580-fig2.tif]
